# Equivalence of the top-down manoeuvre and bottom-up manoeuvre in speed and accuracy of identifying the cricothyroid membrane: a prospective randomised cross-over study

**DOI:** 10.1186/s12873-023-00796-9

**Published:** 2023-03-16

**Authors:** Yohei Kamikawa, Osamu Muto, Hiroyuki Hayashi

**Affiliations:** 1 Department of Emergency Medicine, University of Fukui Hospital, 23-3 Matsuoka Shimoaizuki, Eiheiji-cho, 910-1193 Yoshida-gun, Fukui, Japan; 2 Department of General Medicine, University of Fukui Hospital, 23-3 Matsuoka Shimoaizuki, Eiheiji-cho, 910-1193 Yoshida-gun, Fukui, Japan

**Keywords:** Airway management, Cricothyrotomy, Cricothyroid membrane, Thyroid cartilage, Cricoid cartilage, Academic medical centres, Medical students

## Abstract

**Background:**

Accurate identification of the cricothyroid membrane is crucial for successful cricothyrotomy; however, a manoeuvre that helps identify it both accurately and quickly remains unclear. The effectiveness of the so-called ‘bottom-up manoeuvre’ has never been investigated. This study aimed to examine whether the bottom-up manoeuvre is as rapid and accurate as the conventional ‘top-down manoeuvre’ at identifying the cricothyroid membrane.

**Methods:**

This study was a prospective randomised cross-over trial conducted at an academic medical centre between 2018 and 2019. Fifth-year medical students participated. The students were trained in the use of either the top-down manoeuvre or the bottom-up manoeuvre first. Each student subsequently performed the technique once on a volunteer. The students were then taught and practiced the other manoeuvre as well. The accuracy of cricothyroid membrane identification and the time taken by successful participants only were measured and compared between the manoeuvres using equivalence tests with two one-sided tests.

**Results:**

A total of 102 medical students participated in this study and there was no missing data. The accuracy of identification and time required for success were similar between the top-down manoeuvre and the bottom-up manoeuvre (65.7% vs. 70.6%, taking 13.8 s [interquartile range (IQR): 9.4–17.5] vs. 15.5 s [IQR: 11.5–19.9], respectively). The success rate was statistically equivalent (rate difference, 4.9%; 90% confidence interval [CI], -5.8 to 15.6; equivalence margin, -20.0 to 20.0). The time required for success was also statistically equivalent (median difference, 1.7 s; 90% CI, -0.2 to 3.3; equivalence margin, -4.0 to 4.0).

**Conclusion:**

Among students first trained in both manoeuvres for identifying the cricothyroid membrane, the speed and accuracy of identification were similar between those using the bottom-up manoeuvre and those using the top-down manoeuvre.

## Background

Emergent cricothyrotomy is the ultimate life-saving procedure for patients classified as ‘cannot intubate, cannot oxygenate’ [1]. Successful cricothyrotomy depends on accurate identification of the cricothyroid membrane (CTM) [2, 3]. Misidentification of the CTM may lead to serious complications, such as tracheal wall injury or carotid artery laceration, with incidence rates of 0–40% [2, 4]. The misidentification rate appears to be higher in female and obese patients, based on a study that found a rate of moderate to severe complications of 100% when the subjects had difficult landmarks [2]. The laryngeal handshake was introduced by the Difficult Airway Society in 2015 as the first step in identifying the CTM [1], and the technique especially has an advantage in identifying the midline of the CTM [5]. Although several studies have examined its effectiveness [5–8], a recent meta-analysis revealed that the technique has not demonstrated significant improvement in identifying CTM compared to conventional techniques [9].

CTM identification may be achieved using a palpation technique, which has been described as a ‘conventional technique’, ‘general technique’, or ‘top-down manoeuvre’ [10, 11]. However, the associated success rate is 30–72% [12–15]; with success rates generally being lower (0–39%) in female and obese patients [12, 13, 15–19]. To achieve high accuracy, it has been recommended that the CTM be identified by ultrasound and that a vertical skin incision be performed before a cricothyrotomy is attempted in cases in which the CTM is possibly difficult to palpate [1, 17, 20–22]. Nevertheless, some critics point out that the use of ultrasound takes approximately 1 min more than does the conventional palpation method [2, 6, 17]. Even when a vertical skin incision is performed, palpation methods are still needed to identify the CTM.

The ‘bottom-up manoeuvre’ is informally known by some clinicians but rarely reported. Additionally, the effectiveness of the manoeuvre has never been investigated. This study aimed to examine whether the bottom-up manoeuvre is equivalent to the top-down manoeuvre in accuracy and time required for success among medical students first instructed in both techniques and without any prior cricothyrotomy experience. We hypothesised that the bottom-up manoeuvre was equivalent to the top-down manoeuvre. The present findings may help expand the options for CTM identification.

## Methods

### Study design and setting

This double-blind cross-over study was conducted at an academic medical centre between April 2018 to March 2019 and was implemented as a part of medical student training in the emergency department (ED). The study protocol was approved by the Institutional Ethical Review Board of the University of Fukui Hospital (20,190,019). All study methods adhered to the Declaration of Helsinki and the Consolidated Standards of Reporting Trials Statement.

### Manoeuvres

**Fig. 1:**
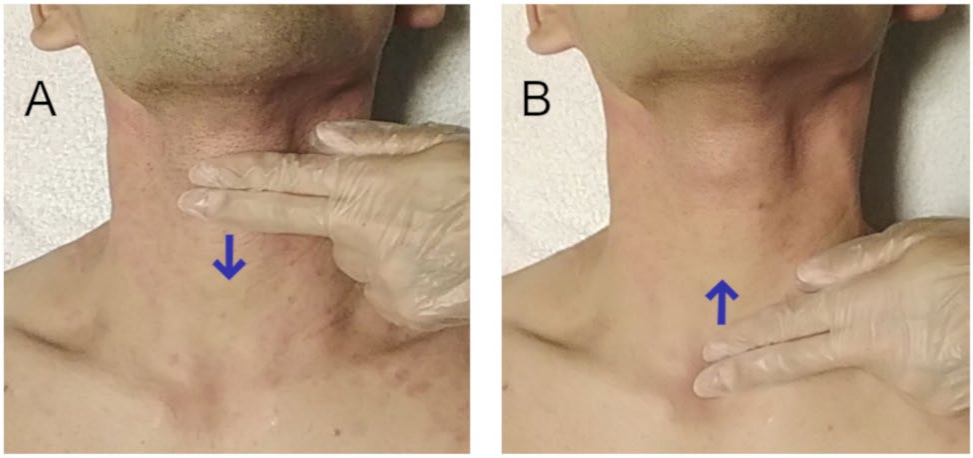
**Schematic representation of the top-down manoeuvre (A) and the bottom-up manoeuvre (B)** Both of the above figures are shown schematically to clarify the difference in the initial position of the fingers during the examination. The fingers are aligned in the sagittal direction in the figures, but practically, the fingers are re-aligned horizontally after this, and then the landmarks described below are carefully explored at a pinpoint with the fingertips. (A) Fingers identify the thyroid cartilage and then slide down over the neck, exploring a cleft corresponding to the cricothyroid membrane. (B) Fingers identify the suprasternal notch and then slide up over the neck, exploring a hard knob corresponding to the cricoid cartilage. The cricothyroid membrane is located beyond the cartilage.

The palpation methods compared in this study are shown in Fig. 1. In the top-down manoeuvre, clinicians stand on the subject’s left side and identify the thyroid cartilage using their fingers, and then slide them along the neck in the caudal direction, locating the CTM cleft. For the bottom-up manoeuvre, clinicians stand on the subject’s left side and put their fingers on the suprasternal notch, and then slide them along the neck in the cranial direction, identifying a hard knob structure, which is the cricoid cartilage; the CTM is found beyond this cartilage [10].

### Selection of participants

All fifth-year medical students at the centre were eligible for this study. All provided written informed consent to participate and signed confidentiality agreements to prevent the study protocol from being leaked to other students. Students who had previously learned or performed cricothyrotomy, declined consent, or violated the study protocol were excluded. The participating students were not informed about the study aims or outcomes of interest. All data were collected and anonymised.

Fifth-year medical students were divided by the Faculty of Medicine into small teams of two or three members. We used these teams as units and randomly (using computer-generated numbers) assigned them to learn either the top-down manoeuvre (odd numbers) or the bottom-up manoeuvre (even numbers) first, in a ratio of 1:1. This randomisation of the order of instruction and procedures attempted to minimise the impact of learning effects. The study was conducted at the beginning of the one-week ED rotation.

### Measurements

The investigator instructed participants to identify the CTM using an anatomical chart of the neck. First, students were taught either of the techniques. The participants were then directed to palpate their own CTM and to imagine the structure. However, as we did not allow them to touch other person’s CTM, they did no practice identifying the CTM before the examination. This didactic training took approximately 15 min.

Immediately after the lecture, the students took turns in attempting to identify the CTM of the investigator, a male in his ages 35–40 years old with a body mass index (BMI) of 35 kg/m^2^, neck circumference of 46.0 cm, thyromental distance of 10.6 cm, and sternomental distance of 18.2 cm. He was able to extend his neck, and he had no history of neck surgery or intubation. When examined by an ED physician, he required deep palpation of the thyroid cartilage, which was classified as ‘difficult landmark palpation’ [2]. The time required for CTM identification was measured by the other team members using a prepared stopwatch (SW-111WT, DRETEC CO., LTD., Saitama, Japan) in order to minimise observer bias.

The procedure involved participants standing with their hands down on the left side of the subject, who lay in a neutral supine position on a bed. When the team member timing the procedure gave a verbal cue to start, the participants began the manoeuvre. When the participant declared ‘I found’, the timing was stopped. The students kept their fingers at the site, and then they marked the point with a permanent marker which they identified as the CTM site. After that, the accuracy of the location was assessed by another investigator (an eighth-year attending ED physician) using ultrasound (Noblus, Hitachi Medical Corporation, Tokyo, Japan) with the longitudinal technique [23]. The location was considered accurate when the identified site was within the CTM area bounded by the thyroid cartilage and the cricoid cartilage. Afterwards, the mark was removed completely with an alcohol swab. Data were collected on participant characteristics (age [years] and sex), task success/failure, and time required to successfully complete the task.

After all the participants had completed procedures, they took a 10-minute break, and then the other manoeuvre was lectured and performed in the same way.

### Outcomes

The primary outcome was the accuracy in identifying CTM. The secondary outcome was the time required for successful identification, more specifically, data from participants who failed the procedure were excluded when assessing the time required.

### Statistical analysis

Categorical variables were reported as counts and percentages, while continuous variables were reported as medians and interquartile ranges (IQRs). Fisher’s exact test was used to compare categorical variables between the groups, and the Mann − Whitney U test was used to compare continuous variables between the groups.

Equivalence tests with two one-sided tests (TOST) were performed to examine the success rate difference and the median difference of the time required for success between the top-down manoeuvre and bottom-up manoeuvre. In the TOST, equivalency is determined when the 90% confidence interval (CI) of the success rate difference or the median difference of the time required for success is within a predetermined equivalence margin [24]. Previous studies have reported clinical significance in ≥ 25% differences for accuracy [5–8, 11, 15, 16, 18–20, 25, 26] and ≥ 4.1 s differences for the required time [6, 8, 15, 25, 26]. Therefore, in the present study, the margins were set at 20% for accuracy and 4.0 s for the required time. The sample size was calculated based on the equivalence margins mentioned above, the success rate of 72% previously reported in identifying CTM for males [13], the standard deviation of 7.2 s for the time required in identifying the CTM estimated from a previous study [10], a power of 80%, a significance level of α = 0.05, and the hypothesis that there was no difference between the groups. As a result, the sample size was determined to be 87 per group for accuracy and 56 per group for time required [27]. When the dropout rate was expected to be 10%, we finally estimated that the sample size was 97 per group. All statistical analyses were performed using R software (version 3.4.1; The R Foundation, Vienna, Austria).

## Results

### Characteristics of study subjects

A total of 102 medical students, 33 (32%) of whom were female, were included. No student was excluded. The median age was 24 years (IQR: 23–25 years). The success rate was 65.7% using the top-down manoeuvre and 70.6% using the bottom-up manoeuvre, with no significant differences between the two groups with regard to success rates or time required for successful identification (Table 1).

**Table 1:** **Participant performance**

	Top-down manoeuvre	Bottom-up manoeuvre	p-value
Success, N (%)	67/102 (65.7)	72/102 (70.6)	0.55
Time required, s Median (IQR)	14.0 (10.0–19.0)	16.0 (11.5–21.3)	0.06
	N = 102	N = 102	
Time required for success, s Median (IQR)	13.8 (9.4–17.5)	15.5 (11.5–19.9)	0.15
	N = 67	N = 72	

IQR: interquartile range. P-values were calculated using Fisher’s exact test or the Mann − Whitney U test.

“Time required” summarises the time taken by all participants. On the other hand, “Time required for success” summarises the time taken by successful participants only.

### Main results

**Fig. 2:**
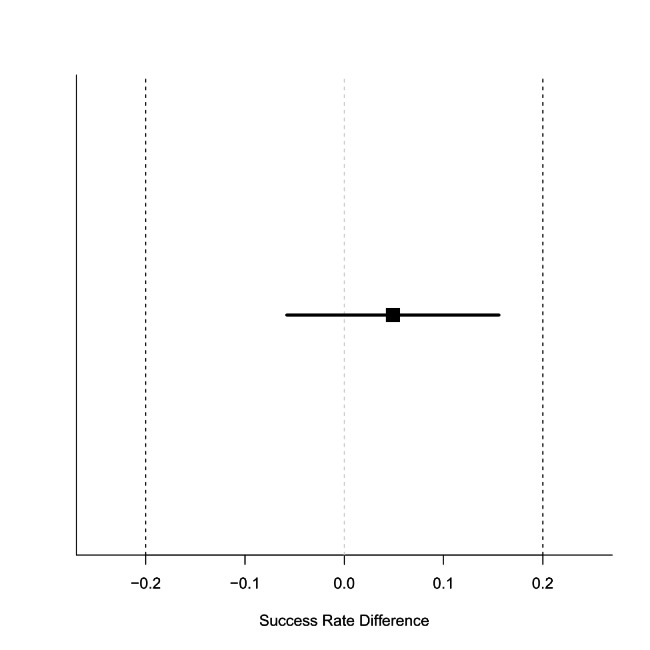
**Equivalency of success rate between the top-down manoeuvre and the bottom-up manoeuvre**

**Fig. 3:**
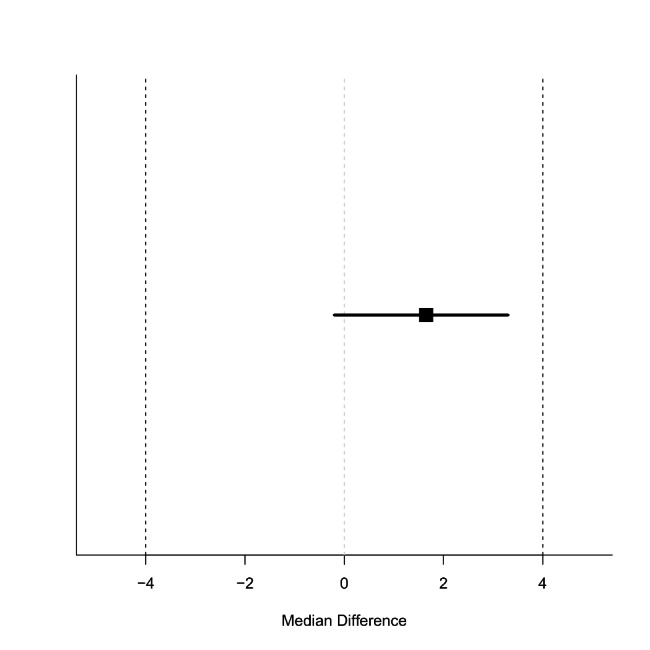
**Equivalency of time required for success between the top-down manoeuvre and the bottom-up manoeuvre**

The TOST for accuracy revealed that the success rate difference between the groups was 4.9% (90% CI: -5.8 to 15.6) (Fig. 2), which was within the 90% CI equivalence margin. Similarly, the TOST for the required time for successful identification revealed that the median difference between the groups was 1.7 s (90% CI: -0.2 to 3.3) (Fig. 3), which was within the 90% CI equivalence margin.

## Discussion

This study demonstrated equivalent accuracy and time required for successful CTM identification between the top-down manoeuvre and the bottom-up manoeuvre, performed by fifth-year medical students instructed newly on these methods and without any prior experience performing cricothyrotomy.

Both the accuracy and the time taken to identify the CTM are similar to those reported in other studies. The accuracy of CTM identification by palpation methods has been estimated to range from 30–72% [12–15], compared with the success rates of approximately 70% in this study. These findings suggest that our instruction was adequate for helping students identify the CTM. Previous studies have reported that the time required to identify the CTM using palpation methods was 8–18 s [10, 25, 28]. The corresponding range in this study was 14–16 s, which is consistent with those previously reported.

These findings suggest that the bottom-up manoeuvre may be effectively used as an alternative to the top-down manoeuvre, expanding the available range of options for CTM identification and improving the safety of cricothyrotomy. Given that the difficulty associated with CTM identification is the primary cause of cricothyrotomy failure and complications [2, 3, 17, 23], training in alternative methods may improve the procedure’s success rates in difficult cases. Medical students and early-career physicians should be trained in both approaches to cricothyrotomy. Nevertheless, it should be noted that the accuracy of the bottom-up manoeuvre, with a success rate of 70.6%, was still as low as that of the top-down manoeuvre. Therefore, training in ultrasound examination and the use of vertical skin incision is necessary as a preliminary preparation for emergency cricothyrotomy in patients with difficult landmark palpation. However, even in cases involving ultrasound examination or skin incision, access to alternative approaches, including the bottom-up manoeuvre, may help physicians verify the location of the CTM, reducing the uncertainty of cricothyrotomy.

In contrast to our findings, Chang et al. [26] reported the effectiveness of a technique named ‘modified upward laryngeal handshake’, which was also a bottom-up approach but slightly different from the manoeuvre used in this study. Notably, they demonstrated that the technique was significantly more accurate than the conventional top-down technique in female patients. The difference between the results of the study by Chang et al. and our study may be attributed to differences between males and females in the structure of the thyroid cartilage. Females have a less prominent thyroid cartilage than males, making it more difficult to detect the CTM using top-down techniques in females than in males [12, 19]. On the other hand, as the anatomy of the cricoid cartilage does not differ significantly by sex, bottom-up techniques palpating the cartilage as the main cue to detect CTM may be particularly useful in female patients. However, more studies are required to further investigate this matter.

This study had some limitations. First, we could not have fully concealed the study protocol, as we did not prohibit other team members from observing the technique of an examinee when they measured the time required for CTM identification. Second, the study results may not be generalisable to clinical populations because the CTM identification was performed on a single person. The person in the role of a patient in this study was a male, who had a high BMI, and his thyroid cartilage was undetectable without deep palpation. He was also able to extend his neck fully. Further research is required to validate the present findings in individuals with other types of body habitus. Third, this study may have omitted the problem of multiple testing because it evaluated two important outcomes, including accuracy and time required. However, since we prioritised the primary and secondary outcomes and reported the results of all the tests performed in this study, we consider that the multiple testing problem was avoided to a certain extent [29]. Fourth, as this study involved medical students, the present findings may not be generalised to experienced physicians. Fifth, this study did not assess the lateral error from the midline of the CTM, and it was certainly insufficient to assess the overall utility of the manoeuvres. Finally, the study did not replicate the real situation of rescuing a dying patient.

## Conclusion

The accuracy and rapidity of the bottom-up manoeuvre in identifying the CTM were equivalent to those of the top-down manoeuvre when performed by medical students with recent training. The bottom-up manoeuvre may be a viable alternative to the standard CTM identification technique. However, further studies are required to determine whether the present findings apply to experienced physicians performing the procedure and to patients of both sexes with different body habitus.

## Data Availability

The datasets generated and/or analysed during the current study are available in the Open Science Framework repository, https://osf.io/5nmgt/ or DOI 10.17605/OSF.IO/5NMGT.

## List of abbreviations

IQR: Interquartile range
CI: Confidence interval
CTM: Cricothyroid membrane
ED: Emergency department
BMI: Body mass index
TOST: Two one-sided test
